# Effects of Carbon Nanotubes/Graphene Nanoplatelets Hybrid Systems on the Structure and Properties of Polyetherimide-Based Foams

**DOI:** 10.3390/polym10040348

**Published:** 2018-03-21

**Authors:** Hooman Abbasi, Marcelo Antunes, José Ignacio Velasco

**Affiliations:** Departament de Ciència dels Materials i Enginyeria Metal·lúrgica, Centre Català del Plàstic, Universitat Politècnica de Catalunya (UPC·BarcelonaTech), C/Colom 114, E-08222 Terrassa, Barcelona, Spain; marcelo.antunes@upc.edu (M.A.); jose.ignacio.velasco@upc.edu (J.I.V.)

**Keywords:** nanocomposites, graphene, carbon nanotubes, hybrid nanoparticles, polyetherimide foams, electrical conductivity, percolation, ultrasonication

## Abstract

Foams based on polyetherimide (PEI) with carbon nanotubes (CNT) and PEI with graphene nanoplatelets (GnP) combined with CNT were prepared by water vapor induced phase separation. Prior to foaming, variable amounts of only CNT (0.1–2.0 wt %) or a combination of GnP (0.0–2.0 wt %) and CNT (0.0–2.0 wt %) for a total amount of CNT-GnP of 2.0 wt %, were dispersed in a solvent using high power sonication, added to the PEI solution, and intensively mixed. While the addition of increasingly higher amounts of only CNT led to foams with more heterogeneous cellular structures, the incorporation of GnP resulted in foams with finer and more homogeneous cellular structures. GnP in combination with CNT effectively enhanced the thermal stability of foams by delaying thermal decomposition and mechanically-reinforced PEI. The addition of 1.0 wt % GnP in combination with 1.0 wt % CNT resulted in foams with extremely high electrical conductivity, which was related to the formation of an optimum conductive network by physical contact between GnP layers and CNT, enabling their use in electrostatic discharge (ESD) and electromagnetic interference (EMI) shielding applications. The experimental electrical conductivity values of foams containing only CNT fitted well to a percolative conduction model, with a percolation threshold of 0.06 vol % (0.1 wt %) CNT.

## 1. Introduction

Polyetherimide (PEI) has recently become popular for use in advanced applications, due to its outstanding combination of high mechanical properties, flame and chemical resistance, and high thermal and dimensional stability. The preparation of PEI-based foams reinforced with carbon-based nanoparticles using water vapor induced phase separation (WVIPS) has shown promising results in terms of homogeneity and filler dispersion [1,2,3,4]. The addition of carbon-based nanofillers to PEI has created a suitable candidate for various advanced applications, such as fuel cells and electromagnetic interference (EMI) shielding [5,6]. Additionally, foaming could facilitate desirable features such as density reduction, damping properties, high thermal insulation, and the potential improvement of electrical conductivity and electromagnetic absorption by promoting wave scattering [7,8,9]. The combination of functional nanoparticles and foaming has a high potential to generate new lightweight composites with high specific strength and multifunctionality [10]. Simultaneous enhancements in electrical and mechanical properties, with the addition of carbon-based nanosized fillers such as graphene nanoplatelets (GnP) or carbon nanotubes (CNT), owing to their high aspect ratio (AR) and exceptional mechanical and electrical properties, have brought important advantages over non-carbon-based nanofillers [11,12,13].

Various attempts to prepare hybrid CNT/graphene materials have been carried out to create transparent conductors [14,15,16,17], electrodes [18], electron field emitters [19], field effect transistors [17], supercapacitors [20], and Li-ion batteries [21,22]. Maxian et al. [23] numerically investigated the electrical percolation behavior of porous systems incorporating carbon 1D- and 2D-fillers assuming perfect random filler distribution and realistically modeling ‘foaming’ by displacing the fillers. Their model was able to successfully capture experimental trends [24] showing the increased conductivity and lower percolation threshold of porous compared to non-porous systems. Sensitivity analysis demonstrated that the electrical percolation behavior of the porous polymer systems incorporating 1D- and 2D-nanofillers was significantly affected by four main factors: (i) porosity level, (ii) type of filler, (iii) filler alignment, and (iv) filler aspect ratio (AR). The type of filler and its AR played the most important role in establishing the percolation threshold.

The present work aimed to extend the applicability of PEI composites by considering the combination of two strategies: foaming of the composites by means of WVIPS and the use of a hybrid nanofiller system based on GnP and CNT. In terms of the first strategy, we have already shown in previous works that WVIPS foaming is an effective method to obtain medium-density PEI foams with homogeneous structures, and that the addition of GnP to PEI and foaming can lead to components with enhanced electrical conductivity. This is crucial in applications requiring high EMI shielding, and especially those where EM absorption mechanisms play a key role, such as in stealth technology [25,26]. On the other hand, it has been shown that hybrid nanofillers based on platelet-like GnP and other conductive nanoparticles such as CNT may promote the formation of an efficient conductive network [27,28,29].

This work considered the preparation of composites based on PEI and different proportions of dispersed CNT (from 0.0 to 2.0 wt %) and GnP (from 0.0 to 2.0 wt %), to give a total nanofillers amount of 2.0 wt %, their foaming by WVIPS, and their characterization in terms of microstructure, cellular structure, thermal stability, viscoelastic behavior and electrical conductivity. We predominantly focused on analyzing how the addition of GnP affects the electrical conduction behavior of the resulting composite foams.

## 2. Experimental

### 2.1. Materials

Thermoplastic polyetherimide (PEI), with the commercial name Ultem 1000, manufactured by Sabic (Riyadh, Saudi Arabia), was used. PEI Ultem 1000 has a density of 1.27 g/cm^3^ and a glass transition temperature (*T*_g_) of 217 °C.

Graphene nanoplatelets, known as GnP (commercial name xGnP M-15 and density of 2.2 g/cm^3^), were supplied by XG Sciences (Lansing, MI, USA). These nanofillers are formed using stacks of graphene nanoplatelets with an average thickness of 6–8 nm and a lateral size of 15 μm, with an approximate surface area of 120–150 m^2^/g and an electrical conductivity of 10^7^ and 10^2^ S/m, respectively, measured parallel and perpendicular to their surface, as reported by the manufacturer.

Multi-wall carbon nanotubes (MWCNT), from now on referred to as CNT, with a carbon content >95%, density of 2.1 g/cm^3^ and characteristic dimensions of 6–9 nm × 5 μm, were purchased from Sigma Aldrich (Saint Louis, MO, USA). These multi-walled carbon nanotubes were prepared by chemical vapor deposition, using cobalt and molybdenum as catalysts.

*N*-methyl pyrrolidone (NMP) was acquired from Panreac Química SA (Barcelona, Spain) with a purity of 99%, a boiling point of 202 °C, and a flash point of 95 °C.

### 2.2. Foam Preparation

Two sets of foams were prepared by WVIPS: a first series of foams containing only CNT (“CNT series”), particularly 0.1, 0.5, 1.0, and 2.0 wt % of CNT; and a second series containing the hybrid nanofillers system through the combination of different amounts of GnP (from 0.0 to 2.0 wt %) and CNT (from 0.0 to 2.0 wt %), for a total nanofiller amount of 2.0 wt % (“Hybrid series”).

A detailed explanation of the WVIPS process is given in previous works [25,26]. In this study, the preparation of foams began with the dispersion of 0.5 g of CNT into 200 mL of NMP, which is known to be a proper solvent for carbon-based suspension at room temperature [30]. High power probe sonication was applied for 60 min using a Fisher Scientific FB-705 ultrasonic processor with a 12 mm solid tip probe at 20% amplitude and 20 kHz output, applying a total amount of energy of 380 kJ (30–60 W), kept at a constant temperature of 50 °C by placing the suspension inside an ice-bath. In the case of the Hybrid series, the corresponding amount of GnP was initially sonicated at 100% amplitude for 30 min, followed by the sonication of CNT in the GnP-NMP suspension. In the following step, PEI was dissolved in the suspension containing the sonicated particles (15.0 wt % PEI solution) at 75 °C and kept stirring at 450 rpm for a period of 24 h. Afterwards, the filler-rich solution was diluted with PEI-NMP (15.0 wt % PEI in NMP) to obtain the 0.1, 0.5, 1.0, and 2.0 wt % CNT-filled composites alongside with the hybrid compositions of 1.5–0.5, 1.0–1.0, and 0.5–1.5 CNT-GnP. The composites corresponding to the Hybrid series (total CNT-GnP amount of 2.0 wt %) are specifically referred to as 2/0, 1.5/0.5, 1/1, 0.5/1.5 and 0/2, with the first number corresponding to the wt % of CNT and the second one to the wt % of GnP.

Subsequently, each solution was poured on a flat glass exposed to air, with an average measured humidity of 75% at room temperature for 4 days, which promoted foaming of the polymer by means of WVIPS. The resulting foams were then washed with a 50/50 mixture of ethanol and water followed by extraction of the remaining solvent, utilizing hot water, stirring at 90 °C for 7 days and intensively drying under vacuum at 140 °C for 7 additional days to fully extract the residual NMP. The typical density of the prepared foams was 0.3–0.5 g/cm^3^, with a final thickness of around 5 mm. Samples were later cut directly from the prepared foams and used in the several characterizations.

### 2.3. Testing Procedure

The density of the foams was measured according to the ISO-845 standard procedure. The porosity of the foam, understood as its void percentage, could be directly obtained from the density values of the foam and respective unfoamed material according to the following expression:

(1) $$Porosity\;(\%)=\left(1-\frac{\rho }{\rho_{s}}\right)\times 100$$

where *ρ* and *ρ_s_* is the density of the foam and density of the solid unfoamed material, respectively.

The morphology of the foams was analyzed using a JEOL (Tokyo, Japan) JSM-5610 scanning electron microscope (SEM). Samples were fractured using liquid nitrogen and a thin layer of gold was sputter deposited onto their surface with a BAL-TEC (Los Angeles, CA, USA) SCD005 Sputter Coater (Ar atmosphere). The values of the average cell size (Φ), cell nucleation density, and cell density (*N*_0_ and *N*_f_, respectively, both in cells/cm^3^) were measured and calculated, respectively, from the analysis of ×300 magnification SEM images using the intercept counting method, a procedure presented in detail in [31]. Five ×300 magnification SEM images were analyzed for each foam. Particularly, *N*_0_ and *N*_f_ were determined assuming an isotropic distribution of spherical cells according to:

(2) $$N_{0}={\left(\frac{n}{A}\right)}^{3/2}\left(\frac{\rho_{s}}{\rho }\right)$$

(3) $$N_{f}=\frac{6}{{\pi \Phi }^{3}}\left(1-\frac{\rho }{\rho_{s}}\right)$$

where *n* is the number of cells counted in each SEM image and *A* is the area of the SEM image in cm^2^. In Equations (2) and (3), *N*_0_ represents the number of cells per volume of unfoamed material and *N*_f_ represents the number of cells per volume of foamed material. For foams that displayed a dual cell size distribution, the proportion of area occupied by each cell population was taken into account when calculating *N*_0_ and *N*_f_.

The analysis of the characteristic (002) diffraction planes of GnP and CNT, as well as the possible crystalline characteristics of PEI in the foams, was carried out by means of wide-angle X-ray diffraction (XRD) using a PANalytical diffractometer (Almelo, The Netherlands) running with CuKα (λ = 0.154 nm) at 40 kV and 40 mA. Scans were performed from 2° to 60° using a scan step of 0.033°.

A TGA/DSC 1 Mettler Toledo (Columbus, OH, USA) STAR System analyzer was used to study the thermal stability of foams, using samples of around 10.0 mg, heating from 30 to 1000 °C at 10 °C/min under a nitrogen atmosphere (constant flow of 30 mL/min) and analyzing the weight loss evolution with temperature.

Thermomechanical analysis was used to study the viscoelastic behavior of foams, particularly their storage and loss moduli (*E*’ and *E*’’, respectively), as well as PEI’s glass transition temperature (*T*_g_). A DMA Q800 from TA Instruments (New Castle, DE, USA) was used in a single cantilever configuration. Samples were analyzed from 30 to 300 °C at a heating rate of 2 °C/min, applying a dynamic strain of 0.02% and frequency of 1 Hz. Rectangular shape specimens were prepared with a length of 35.5 ± 1.0 mm, width of 12.5 ± 1.0 mm, and thickness of 3.0 ± 0.5 mm. Three different measurements were performed for each sample (error < 5%).

Samples of 20 mm × 20 mm × 1 mm were prepared to measure electrical conductivity using a 4140B model HP pA meter/dc voltage source with a two-probe set. The surfaces of the samples in contact with the copper electrode pads were covered with a thin layer of colloidal silver conductive paint, with an electrical resistance between 0.01 and 0.1 Ω/cm^2^ to guarantee perfect electrical contact. A direct current voltage was applied with a range of 0–20 V, voltage step of 0.05 V, hold time of 10 s, and step delay time of 5 s. 

The electrical conductivity (*σ*, in S/m) was calculated using:

(4) $$\sigma =1/\rho_{v}$$

and

(5) $$\rho_{v}=RA_{E.C}/d$$

where *ρ*_v_ (Ω·m) is the electrical volume resistivity, *R* is the electrical resistance of the sample (in Ω), *A*_E.C_ is the area of the surface in contact with the electrode (in m^2^), and *d* is the distance between the electrodes (in m).

Considering that porosity could affect the surface area in contact with the electrode, the cell size and the cell density of foams were used to apply a correction to the values of electrical conductivity (*σ*_corr_) by taking into account variations in effective surface area as follows:

(6) $$\sigma_{\mathrm{corr}}=\frac{d}{R(A_{\mathrm{non-cell}}+A_{\mathrm{cell-hemisphere}})}\;$$

where *A*_non-cell_ is the *A*_E.C_ with the cell section area excluded and

(7) $$A_{\mathrm{cell-hemisphere}}=\left(\frac{n}{A}\right)A_{E.C}\left(2\pi {\frac{\Phi }{4}}^{2}\right)$$

Therefore:

(8) $$A_{\mathrm{non-cell}}+A_{\mathrm{cell-hemisphere}}=A_{E.C}+\left(\left(\frac{n}{A_{}}\right)A_{E.C}\left(\pi {\frac{\Phi }{4}}^{2}\right)\right)$$

the values of *n*, *A*, and Φ were obtained by analyzing SEM micrographs, and represent the number of cells, the corresponding area of the micrograph, and average cell size, respectively.

## 3. Results and Discussion

### 3.1. Cellular Structure

The composition of both series of foams and respective densities are presented in Table 1. As can be seen, foam density was affected by the addition of CNT, as density grew higher with increasing CNT concentration.

**Figure 1:**
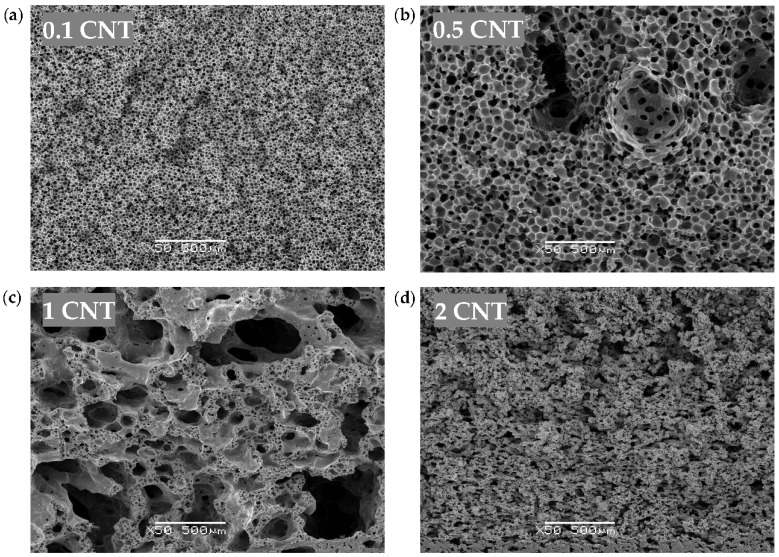
Typical SEM images at ×50 magnification showing the general cellular structure of the CNT series foams: (**a**) 0.1 wt %, (**b**) 0.5 wt %, (**c**) 1.0 wt %, and (**d**) 2.0 wt % of CNT.

**Figure 2:**
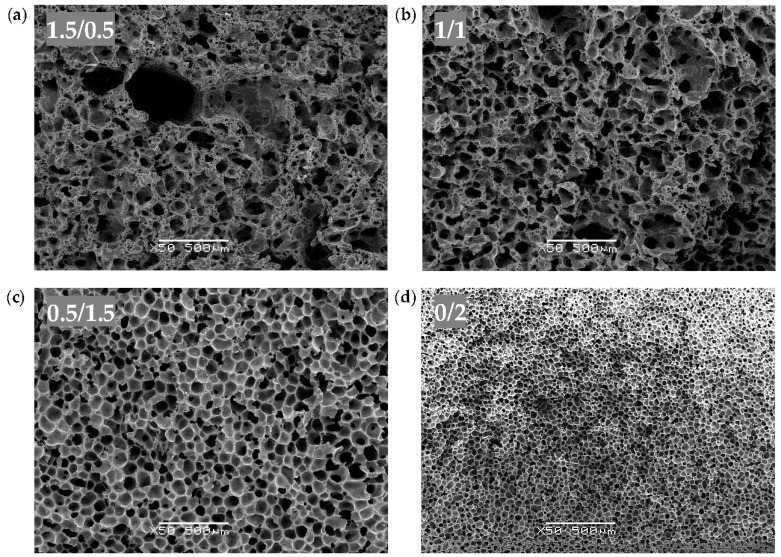
Typical SEM images at ×50 magnification showing the general cellular structure of the Hybrid series foams: (**a**) 1.5 wt % CNT and 0.5 wt % GnP, (**b**) 1.0 wt % CNT and 1.0 wt % GnP, (**c**) 0.5 wt % CNT and 1.5 wt % GnP, and (**d**) 0.0 wt % CNT and 2.0 wt % GnP.

The characteristic micrographs showing the general cellular structure of both the CNT and Hybrid series are, respectively, presented in Figure 1 and Figure 2. The morphological characteristics of all the samples are compiled in Table 2.

The cellular structure of the CNT series foams changed from homogeneous closed-cell (0.1 CNT) to homogeneous porous inter-connected (2 CNT). Foams with intermediate CNT concentrations (0.5 CNT and 1 CNT) showed complex cellular structures. Sample 0.5 CNT displayed a quasi-unimodal structure, with slightly inter-connected cells, bigger than those of the 0.1 CNT foam. Sample 1 CNT developed a dual cell size population of partially inter-connected cells with very different cell sizes.

Significant differences were observed in terms of the cellular structure of the foams with varying CNT content, which could be the consequence of two main simultaneous effects of CNT during cell formation and growth. On the one hand, CNT interfered with solvent/non-solvent exchange, slowing it down. As a result, the cell nucleation rate decreased, what leads to increased cell sizes, as observed up until 1 wt % CNT. On the other hand, due to the strong surface interaction between CNT and PEI, as the amount of CNT increased the mobility of the polymer decreased in the solution, that is, the CNT surface strongly limited polymer mobility, favoring the formation of inter-connected cells/porous structure. Due to this effect, the inter-connected cell structure of the foams increased with the CNT content up until 2 wt % CNT.

In the 1 CNT foam, a dual cell structure developed as a consequence of the two different cell nucleation stages. As the first formed cells grew, the remaining solution got richer in polymer due to the extraction of the solvent, leading to the nucleation of a second cell population. It has recently been reported how polymer concentration plays a key role in the resultant cell size of foams prepared by WVIPS [32].

In the particular case of the 2 CNT foam, the high restriction to polymer mobility prevented the formation of cells, resulting in a generalized inter-connected porous structure. In this case, the solvent exchange with water was not confined within the cells and, rather, expelled from the foam through the formed porous structure.

The foam porosity decreased with increasing CNT and, as mentioned before, the foam density increased with increasing CNT content. The cause behind the increased density and decreased porosity of the foams containing the higher CNT concentrations (1 wt % CNT and 2 wt % CNT) seems to be due to a collapse of the inter-connected cell structure. 

The Hybrid series foams displayed increased cellular structure homogeneity with a decreasing concentration of CNT (see Figure 2), which coincides with the results obtained for the CNT series. As can be seen in Figure 2a,b, Hybrid series foams with a higher concentration of CNT (1.5/0.5 and 1/1 samples) showed a dual cell size distribution because of two different cell nucleation steps. Additionally, comparing the SEM images of the 0.5/1.5 and 1/1 hybrids (Figure 2b,c) with the images of 0.5 CNT and 1 CNT (Figure 1b,c), it can be seen that the addition of GnP provided further cellular structure homogeneity.

As in the CNT series, the average cell size in the Hybrid series displayed a general positive trend along with the increasing content of CNT, which was the direct result of CNT slowing down the solvent exchange process.

The porosity of the Hybrid series also showed a general decreasing trend with increasing CNT content, as well as an increasing trend in density. As can be seen, the influence of GnP on the morphology of the Hybrid series foams was less relevant than CNT.

**Figure 3:**
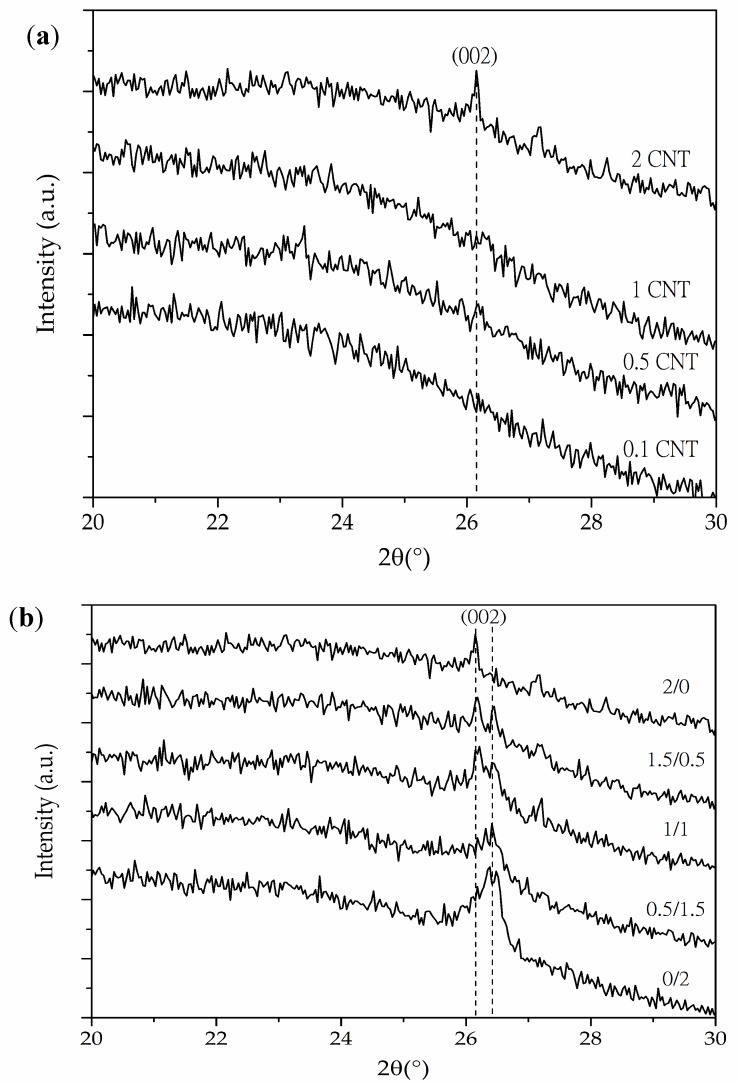
X-ray spectra of (**a**) CNT series foams and (**b**) Hybrid series foams showing the characteristic (002) diffraction plane of CNT and the (002) diffraction plane of CNT and GnP, respectively.

As can be seen in Figure 3a, the X-ray spectra of the CNT series foams demonstrated the proper dispersion and possible exfoliation of the nanotubes throughout PEI’s matrix, as foams with 0.1, 0.5, and 1.0 wt % of CNT showed an absence of the (002) diffraction plane found at 2θ = 25.8°, characteristic of CNT. The disappearance of this peak was related to the dispersion and partial exfoliation of the nanotubes promoted by the combined effects of high power sonication and later foaming. On the other hand, as seen in Figure 3b, some of the Hybrid series foams presented two peaks at 25.8° and 26.5°, respectively corresponding to the characteristic (002) crystal plane of CNT and GnP. This dual peak was observed for 1.5/0.5 and 1/1 foams, which could indicate the absence of total exfoliation of nanoparticles throughout PEI’s matrix. These results reflect one of the potential causes of the enhancement of electrical conductivity for these composites, due to the formation of an effective conductive network by physical contact between GnP and CNT.

### 3.2. Thermal Stability

**Figure 4:**
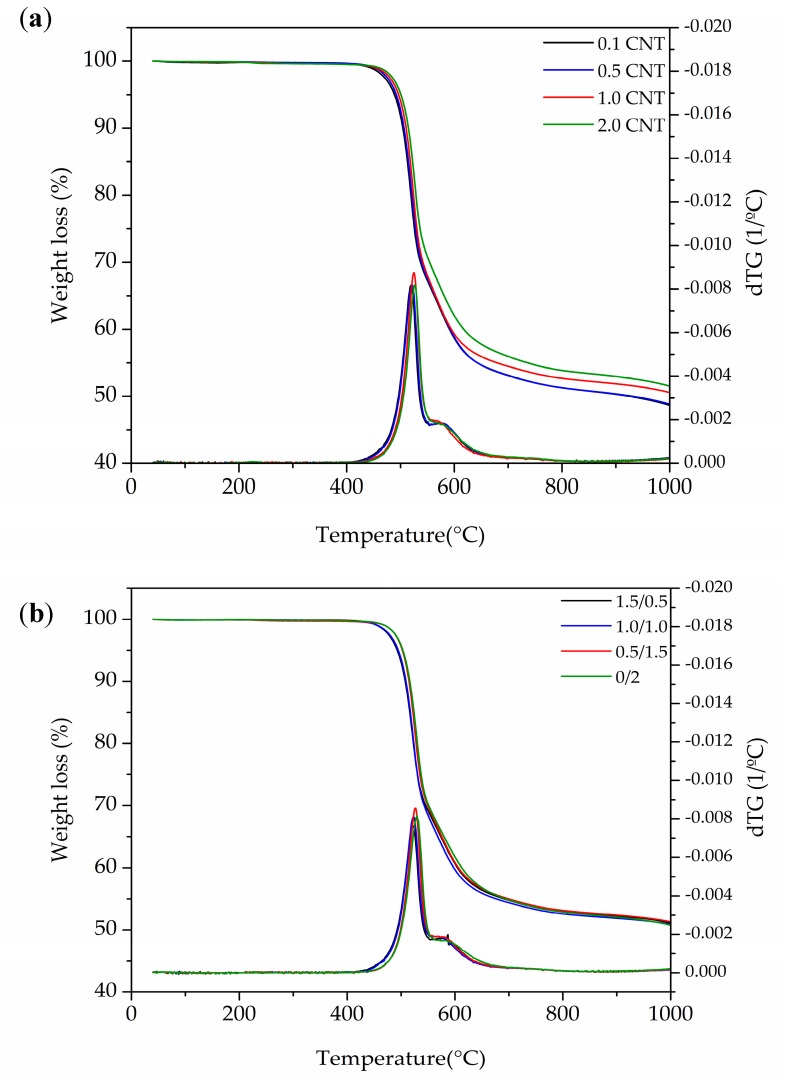
TGA and DTG thermograms of (**a**) CNT series foams, and (**b**) Hybrid series foams.

Thermogravimetric analysis of the foams demonstrated a decomposition retardancy that was induced by increasing the concentration of CNT (see the thermograms shown in Figure 4 and the results presented in Table 3). This could be attributed to the fact that the carbon nanotubes seemed to favor a better physical barrier in the prepared foams or a higher thermal conductivity, facilitating heat dissipation and, therefore, avoiding the accumulation of heat at a certain point [33,34]. Additionally, the combination of GnP and CNT resulted in a more complex decomposition behavior. GnP nanoparticles seemed to have more influence on delaying foam degradation, which could be expected from the barrier effect of GnP induced by its layered-shape hindering the escape of volatile gases. As a consequence, by increasing the concentration of CNT in the Hybrid series, foams showed faster degradation. It has been reported [35,36] that layered-shaped particles could promote a barrier effect by increasing tortuosity and delaying the discharge of volatile products, therefore slowing down the decomposition process. 

The influence of the additional CNT on the morphology of the foams (see Section 3.1 Cellular Structure) could have also had an effect in altering the velocity of decomposition by increasing the surface area of cells and their interconnectivity.

As can be seen in Table 3, a delay of around 10 °C was observed when the concentration of CNT increased from 0.1 to 2.0 wt %. Later, the decomposition followed this behavior with a steeper trend, reaching around 25 °C of delay for a 40 wt % loss (maximum temperature value around 617 °C for the sample with 2.0 wt % CNT). The Hybrid series did not follow the same trend. As mentioned before, foams with a high amount of GnP could have benefited from the layered-shape of the particles by delaying decomposition. Particularly, the 0.5/1.5 Hybrid series foam seemed to exceed the sample with 2.0 wt % of CNT at the onset temperature of decomposition by a close margin, slowly falling behind at more advanced stages of thermal decomposition. Increasing the concentration of CNT in the Hybrid series foams did not seem to enhance thermal stability when compared to the initial sample with only 0.5 wt % of CNT.

### 3.3. Viscoelastic Behavior

**Figure 5:**
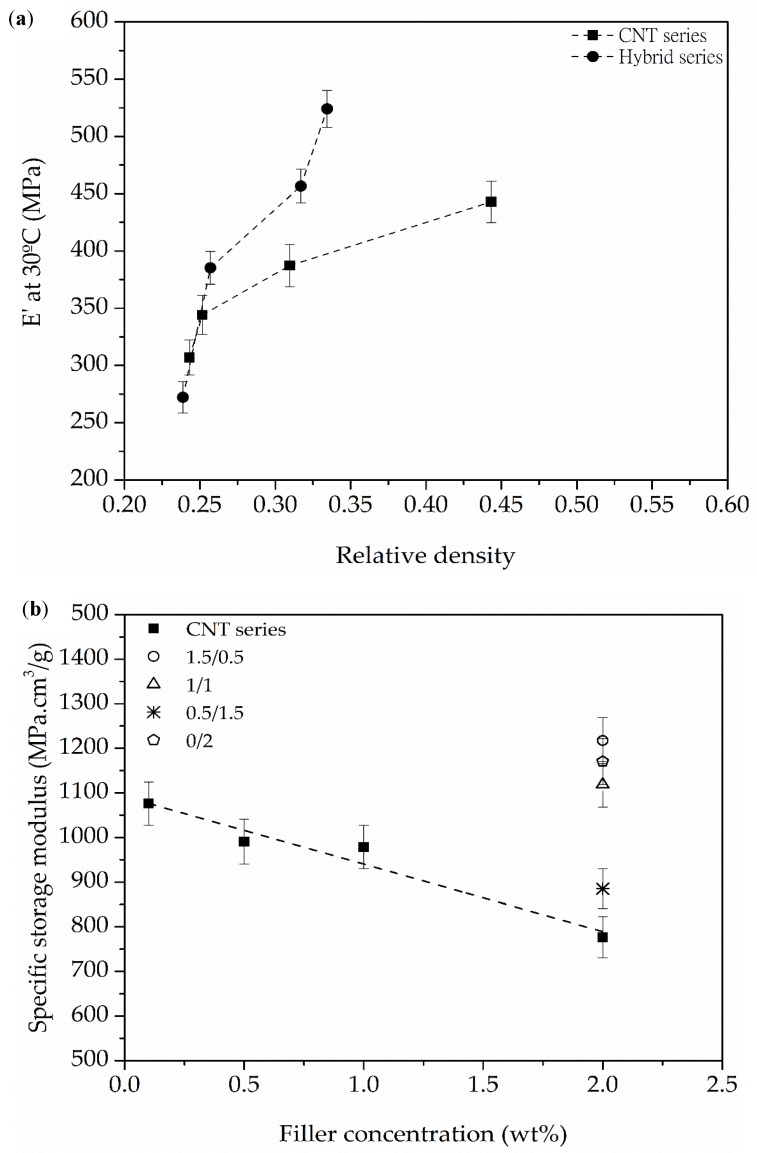
(**a**) Evolution of the storage modulus (*E*’) measured at 30 °C with increasing relative density and (**b**) evolution of the specific storage modulus at 30 °C with increasing filler concentration for CNT series foams and Hybrid series foams.

Thermomechanical analysis of the foams showed that two main factors affected their viscoelastic behavior: the density and cellular structure of the foams, and the concentration of CNT. Both factors are closely related, as the amount of CNT had a clear direct effect on the final cellular structure of the foams (see Section 3.1 Cellular Structure). As can be observed in Figure 5a, the highest measured value of *E*’ at 30 °C, directly related to the behavior of the elastic portion of the material, corresponded to the foam with the highest amount of CNT in both series (2.0 wt % in CNT series and 1.5 wt % in Hybrid series). This was related to a higher foam density induced by the presence of CNT.

The foam’s structural influence on mechanical behavior could be extracted from the changes observed in the specific storage modulus of the foams (Figure 5b). The results showed that the morphological differences caused by increasing the amount of CNT could result in a counter effect, reducing the specific storage modulus of CNT series foams. However, the addition of GnP and its mentioned effect on the cellular structure in the Hybrid series foams resulted in higher values of the specific storage modulus, opening up a possible strategy to exploit the mechanical reinforcing effects of these carbon nanoparticles for these type of foams.

With respect to the viscous response, results presented in Table 4 suggest that nanofillers could have opposing effects on the viscous behavior of the foams. On the one hand, a lubricating effect facilitates the mobility of PEI molecules surrounding the nanofillers and, on the other, there is a restrictive element to molecular mobility due to an enhanced surface interaction with the polymer molecules. CNT series foams showed a 1–3 °C decrease in the maximum temperatures of both tan δ and *E*’’ when increasing the amount of CNT from 0.1 to 2.0 wt %. Considering that these values reflect PEI’s *T*_g_, the decreases seem to be related to higher molecular mobility when increasing the concentration of CNT. There are several studies suggesting an increase in molecular mobility is a consequence of CNT addition [34,37]. Liu et al. [37] suggested that MWCNT have self-lubricating properties, as they are formed by sp^2^ bonded cylindrical layers that can easily slide and move upon each other as inter-layer interaction is controlled by weak Van der Waals forces. 

Other works have shown that nanofillers can act to restrict molecular mobility due to the high and strong interfacial interaction that can be established between nanometric-sized particles, such as carbon nanotubes or graphene, and polymer molecules [38,39]. As can be seen in Table 4, Hybrid series foams did not show any clear tendency related to the two previously mentioned factors. 

The intensity of the characteristic peak of the *T_g_* in the *E*’’ curve showed clear increases when augmenting the concentration of CNT in the CNT series foams from 0.1 to 2.0 wt %, indicating an increase in the amount of the viscous response of the foams. On the other hand, Hybrid series foams showed that the combination of CNT and GnP could induce a reduction in the viscous response when compared to foams having the same amount of either nanofiller alone. The increase in the intensity of tan δ could be associated with the energy damping response of the foams, as the dual structure in the CNT series seemed to play a role in dissipating energy alongside the presence of a higher amount of CNT. However, the 2.0 wt % CNT foam presented a low value of tan δ, which could be related to its particular cellular structure, formed by inter-connected open pores. As Hybrid series foams displayed more homogeneous cellular structures when increasing the concentration of GnP, they showed an increase in tan δ.

Variations in the width of the loss modulus and tan δ peaks corresponding to the *T*_g_ were quantified by measuring their FWHM (see values presented in Table 4). A slight decrease was observed in terms of the FWHM measured in the loss modulus (*E*’’) for CNT series when increasing the amount of CNT, while for Hybrid series the FWHM decreased when the GnP concentration increased. It has been demonstrated by several authors that a lower FWHM value of the loss modulus peak is indicative of higher molecular relaxation [40,41]. Comparatively, as the decrease in the FWHM was lower for the CNT series when increasing the amount of CNT, these foams displayed a lower restriction to molecular relaxation when compared to the Hybrid series. The addition of CNT-GnP hybrids seemed to restrict chain segment relaxation, as Hybrid series foams presented globally higher FWHM values measured in the loss modulus when compared to CNT series (besides globally higher peak intensities). In terms of tan δ, both CNT and Hybrid series presented significant FWHM increases with rising CNT concentration, related to efficient PEI restriction by the nanotubes, which acted by avoiding localized strains [42]. This could be another factor assisting energy dissipation in these foams. Additionally, Hybrid series foams showed more pronounced energy dissipation as a result of a synergic effect between CNT and GnP, which disappeared in the sample containing only GnP.

### 3.4. Electrical Conductivity

The porosity of both CNT series foams and Hybrid series foams was taken into account when calculating their effective surface area and was used to determine a corrected value of electrical conductivity (σ_corr_). Calculations showed that the corrected values could drop to around half when considering the presence of a porous surface (see values presented in Table 5). These corrected values have been used in further discussions.

**Figure 6:**
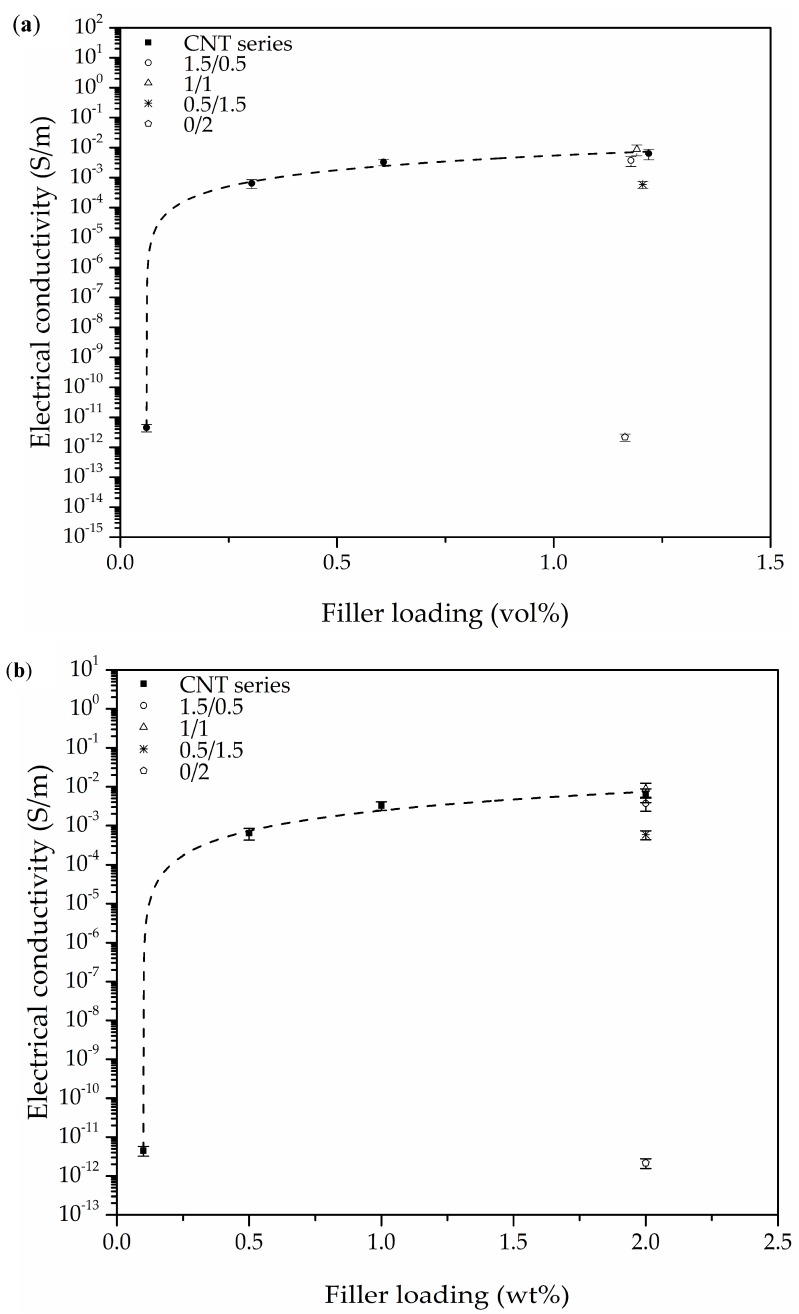

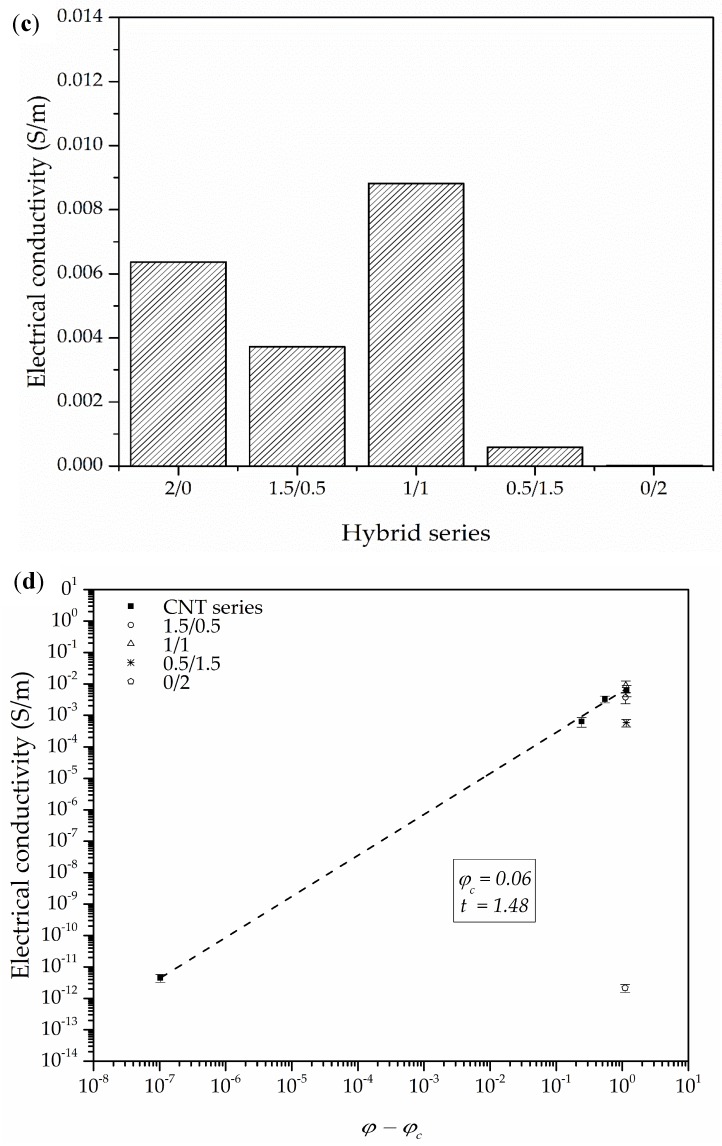
Electrical conductivity evolution with filler loading ((**a**) vol % and (**b**) wt %) for CNT series foams and Hybrid series foams; (**c**) comparison of the electrical conductivity of Hybrid series foams; and (**d**) evolution of electrical conductivity with reduced filler loading (ϕ − ϕ_c_*)* assuming a percolative conduction model.

The electrical conductivity results presented in Figure 6 illustrate the significant influence of the CNT-GnP hybrid network in inducing higher electrical conductivity when compared to foams containing only CNT. Among Hybrid series foams, the 1/1 hybrid foam was the one that displayed the highest value of electrical conductivity, which was related to the formation of an optimum conductive network within the cell struts of PEI for electrical conduction. 

**Figure 7:**
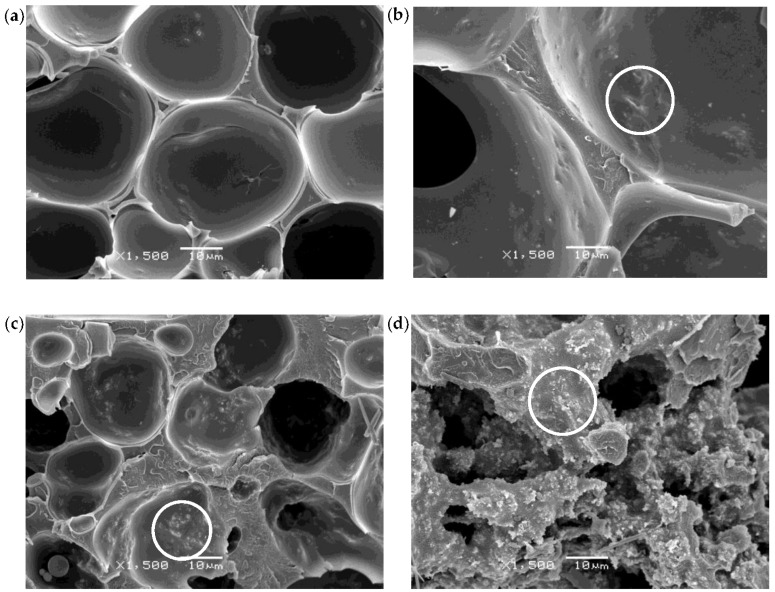
High magnification SEM images of CNT series foams: (**a**) 0.1% CNT; (**b**) 0.5% CNT; (**c**) 1.0% CNT; and (**d**) 2.0% CNT. White circles show physical contact between CNT.

**Figure 8:**
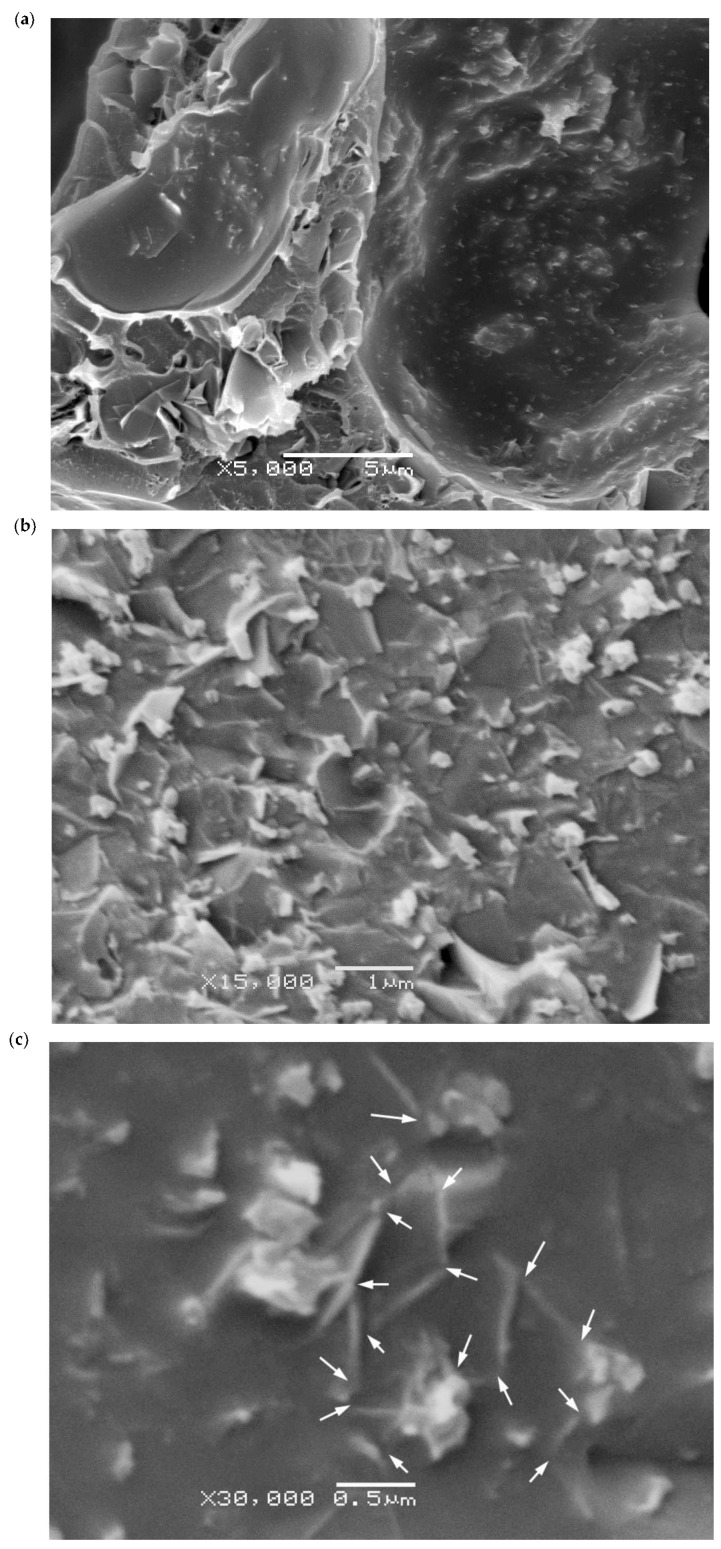
(**a**–**c**) Characteristic high magnification SEM images showing nanoparticle dispersion in Hybrid series foams. White arrows in (**c**) show physical contact between nanoparticles.

As previously mentioned, the X-ray spectra of the Hybrid series foams (Figure 3b) illustrated the appearance of two peaks corresponding to the (002) diffraction plane of CNT and GnP that could indicate the incomplete exfoliation of nanofillers. However, a good distribution of the nanoparticles resulted in the formation of a proper conductive network. Moreover, as seen in the high magnification micrographs presented in Figure 7 and Figure 8, a certain level of physical contact between CNTs was obtained within the cell walls, which induced electrical conduction through the formation of an effective percolative network. This physical contact between nanofillers was more evident in the Hybrid series foams, with the 1/1 hybrid foam apparently displaying an ideal distribution of nanofillers in the cell struts in terms of forming an effective conductive pathway (Figure 8).

As can be seen in Figure 6a, CNT series foams displayed increasingly higher values of electrical conductivity when increasing the amount of CNT up to 2.0 wt % (equivalent to 1.22 vol %). As shown, when increasing the amount of CNT from 0.1 to 0.5 wt % (0.06 to 0.30 vol %), electrical conductivity significantly improved from 4.5 × 10^–12^ to 6.4 × 10^–4^ S/m. The 1/1 hybrid foam displayed even greater electrical conductivity of 8.8 × 10^–3^ S/m, positioning itself as one of the highest registered electrical conductivity measurements for polymer-based foams, with only 2.0 wt % of conductive fillers (see Figure 6c). This could be explained by two causes assisting the formation of an effective conductive network: firstly, high power sonication has been proven to have a large influence on enhancing the dispersion level of carbon-based nanofillers in liquid suspensions; and secondly, the combination of GnP and CNT exhibited a synergic effect due to high individual AR levels and their impact on the cellular structure of the resulting foams. A number of works have achieved relatively low percolation thresholds for unfoamed composites with the addition of hybrid carbon-based conductive nanoparticles [27,28,29]. Additionally, Wu et al. [43] conducted a study using CNT and carbon black (CB) as hybrid fillers for a biodegradable polylactide composite, showing the synergic effect between both fillers in controlling cell size and forming an effective network, significantly enhancing the electrical conductivity of the foamed composites, especially when compared to similar foams containing only CNT. Maxian et al. [23] also studied the combination of GnP and CNT using a new numerical model considering nanofiller random distribution in a porous polymeric matrix in order to predict the electrical percolation behavior of polymer-based composites. In their simulations, the hybrid system exhibited significantly lower percolation values in porous systems when compared to the prediction given by the rule of mixtures, demonstrating the synergic effects of combining CNT and GnP.

A tunneling conduction mechanism has been considered as a suitable model for various polymer foams containing carbon-based nanoparticles, with a range of nanofiller concentrations before the formation of a continuous conductive path [25,40,41]. Initially, a tunnel-like conduction mechanism was considered for both CNT and Hybrid series foams, but no linear trend was observed due to the high electrical conductivity of foams with a nanofiller concentration above 0.1 wt %. Therefore, a percolative conduction model was used, as the nanofiller content was enough to establish an electrical percolation network. The percolative model has been used extensively for polymeric composites containing CNT as a conductive nanofiller [23,43,44,45,46,47,48]. 

In a percolative model, the electrical conductivity (*σ*) above a certain critical concentration (ϕ_c_), commonly called the percolation threshold, is given by:

*σ* ∝ (ϕ − ϕ_c_)*^t^* (9)

where ϕ is the volume fraction of particles in the material and *t* is the percolation exponent [2,49]. As the filler content increases above the threshold value, conductivity rises drastically, indicating the formation of a conductive path. As presented in various cases, the critical exponent *t* is commonly assumed to depend on particle dimensionality, with calculated values of around *t* ≈ 1.3 and *t* ≈ 2.0 corresponding to two and three dimensional systems, respectively [48,50,51,52]. The results of electrical conductivity as a function of nanofiller loading are presented in Figure 6a,b for all foams, with a dash line illustrating the fit to Equation (9). The fitting curve gave a value of the percolation threshold of ϕ_c_ = 0.061 vol % (0.1 wt %) and an exponent of *t* = 1.48 (Figure 6d). As can be seen, the percolation threshold almost overlaps with the CNT concentration corresponding to the CNT series foam having the lowest CNT amount (0.1 wt %). This could indicate that the precise calculation of the percolation threshold requires increasing the number of foams with compositions near this value. Additionally, for a distribution of particles, the excluded volume concept gives the following relation between the percolation threshold and the AR of the nanofiller(s) [53]:

ϕ_c_ ≈ 1/η (10)

where the analysis indicates that most solid composites seem to provide experimental values similar to those that were theoretically predicted when the particles are homogeneously distributed in the composite. For randomly oriented tubular-like particles such as carbon nanotubes:

η = *L*/*W* (11)

with *L* and *W* representing the length and diameter of the particle, respectively. The calculated theoretical value of the threshold was based on the average size of CNT obtained from high magnification micrographs (1 μm ≥ *L* ≥ 0.2 μm) and the assumption that the diameter of the nanotubes remained constant after sonication (*W* = 0.0075 μm, as indicated by the manufacturer). The obtained value of ϕ_c_ was estimated to be ≤0.1 wt % (0.01–0.04 vol %), slightly behind the experimentally calculated value. Assuming the presence of well-dispersed particles after ultrasonication, the theoretical calculation seems to show that the percolation threshold could be even lower than estimated for this particular system. 

While a value of *t* = 1.48 seems to suggest the existence of a two dimensional charge transport system, various studies with obtained *t* values around 1.3 have claimed otherwise [52,54]. Bauhofer and colleagues [48] conducted a review of over 147 experimental studies on the electrical percolation of polymer composites with CNT, concluding that the value of *t* could not be related to any dimensional parameter and, therefore, no well-founded conclusion about CNT network geometry could be provided from most of the experimentally achieved values of *t*.

The electrical conductivity values showed that these foams could fulfil the requirements necessary for applications such as electrostatic discharge (ESD) and electromagnetic interference (EMI) shielding with conductive filler concentrations as low as 2.0 wt %. Various studies have shown that EMI shielding efficiency depends on many factors, including the electrical conductivity of the material [55,56,57]. EMI shielding materials are required to have an electrical resistance below 10^5^ Ω, while the range for materials for ESD applications falls between 10^12^ and 10^5^ Ω [58]. Our previous studies have shown that foaming can also enhance EMI shielding efficiency in cases referring to PC-based nanocomposites [8,9]. Therefore, the high conductivity of these foams could make them suitable candidates for EMI shielding and ESD applications. 

## 4. Conclusions

In terms of cellular structure, the addition of CNT resulted in PEI foams with distinctive structures depending on the amount of CNT: a homogeneous unimodal distribution of closed cells (0.1 wt % CNT), a heterogeneous dual distribution with both closed as well as inter-connected pores (1.0 wt % CNT), and a homogeneous unimodal distribution of inter-connected open pores (2.0 wt % CNT). A similar tendency was observed for CNT-GnP Hybrid series foams and a similar dual structure was formed with both smaller pores and closed cells by adding 0.5 wt % GnP, while keeping 1.5 wt % CNT. Altering the composition to a 1/1 ratio of CNT and GnP resulted in an increase in the homogeneity of the structure, with both bigger closed cells and inter-connected pores, showing that the addition of GnP favored the formation of a finer and more homogeneous cellular structure. These different cellular structures resulted as a consequence of at least two combined effects: (a) the effect of CNT on the kinetics of solvent/non-solvent exchange, and (b) the effect of CNT on the mobility of the polymer. The studied systems were sufficiently complex and demand further investigation in order to elucidate the responsible mechanisms behind the formation and evolution of the cellular structure and the influence of each type of nanoparticle.

CNT series foams displayed an increasingly higher decomposition retardancy when increasing the amount of CNT, while the addition of GnP in combination with CNT seemed to have a high influence in delaying foam degradation, as expected from the more effective barrier effect of layered graphene in hindering the escape of volatile gases during combustion.

Two main factors were found to affect the viscoelastic behavior of the foams: their density and cellular structure and the amount of CNT, given that, indirectly, the amount of CNT also had an important effect in setting the final cellular structure characteristics of the foams. As expected, foams that displayed the highest values of storage modulus were the ones with the highest amount of CNT, related to a higher reinforcing effect of CNT when compared to GnP. Meanwhile, the heterogeneity and existence of open pores, as a consequence of CNT incorporation, resulted in lower values of the specific storage modulus when compared to foams with a lower amount or without CNTs. The incorporation of GnP in the Hybrid series foams and its effect on the formation of a more homogeneous cellular structure resulted in a rise in the specific storage modulus.

Regarding the viscous response, while the addition of only CNT led to foams with lower glass transition temperatures, related to a higher mobility of PEI molecules due to a lubricating effect of CNT, the combination of GnP and CNT did not significantly affect the viscous response of PEI.

All values of measured electrical conductivity were corrected taking into account the porosity of foams and their respective effective surface area. Although foams containing only CNT already displayed high electrical conductivity values, comparatively, the combination of 1.0 wt % GnP and 1.0 wt % CNT resulted in the foam with the highest electrical conductivity (8.8 × 10^–3^ S/m). This was related to the formation of an optimum conductive network within the cell struts of PEI for electrical conduction by physical contact between particles, as assessed by X-ray diffraction and the analysis of high magnification micrographs. This value is one of the highest electrical conductivities registered so far for polymer-based foamed systems containing carbon-based conductive nanofillers. The experimental results fitted well to a percolative conduction model, with a percolation value threshold as low as 0.06 vol % (0.1 wt %) CNT, indicating that the combination of GnP and CNT formed an effective network for electrical conduction. 

Due to the combination of high electrical conductivity and reduced density, these foams can target sectors such as telecommunications and aerospace for applications related to ESD and EMI shielding.

## Figures and Tables

**Table 1:** Composition of the foams and their respective densities.

Series	Sample code	Density	CNT (wt %)	GnP (wt %)	Total filler (vol %)
Carbon nanotubes (CNT)	0.1 CNT	0.32	0.1	0.0	0.06
	0.5 CNT	0.31	0.5	0.0	0.30
	1 CNT	0.40	1.0	0.0	0.61
	2 CNT	0.57	2.0	0.0	1.22
Hybrid	1.5/0.5	0.43	1.5	0.5	1.18
	1/1	0.41	1.0	1.0	1.19
	0.5/1.5	0.31	0.5	1.5	1.21
	0/2	0.33	0.0	2.0	1.16

**Table 2:** Cellular structure characteristics, average cell sizes, and cell densities of the CNT and Hybrid series foams.

Sample code	Homogeneity	Cell type	Porosity (%)	Cell size * (μm)	Cell density (Cells/cm^3^)	
					*N*_f_	*N*_0_
0.1 CNT	Homogeneous (unimodal)	Closed	74.8	Low 23.4 (6.7)	1.1 × 10^8^	4.4 × 10^8^
0.5 CNT	Homogeneous (quasi-unimodal)	Slightly inter-connected	75.7	Medium 55.3 (19.9)	8.5 × 10^6^	3.2 × 10^7^
1 CNT	Heterogeneous (dual)	Partially inter-connected	69.0	High 194.6 (41.1)	1.2 × 10^7^	5.2 × 10^6^
				Low 17.7 (8.3)		
2 CNT	Homogeneous (unimodal)	Inter-connected	55.7	Low 26.9 (19.3)	5.4 × 10^7^	5.1 × 10^7^
1.5/0.5	Heterogeneous (dual)	Partially inter-connected	66.6	High 81.0 (29.1)	5.3 × 10^7^	1.5 × 10^7^
				Low 14.2 (10.1)		
1/1	Heterogeneous (dual)	Partially inter-connected	68.3	High 84.6 (37.0)	9.5 × 10^7^	1.7 × 10^7^
				Low 15.6 (9.1)		
0.5/1.5	Homogeneous (unimodal)	Closed	76.1	Medium 71.2 (24.0)	4.0 × 10^6^	8.8 × 10^6^
0/2	Homogeneous (unimodal)	Closed	74.3	Low 33.2 (10.4)	3.9 × 10^7^	1.2 × 10^8^

* Cell size standard deviation is presented between parentheses.

**Table 3:** Thermogravimetric results for CNT series foams and Hybrid series foams.

Series	CNT (wt %)	GnP (wt %)	Decomposition temperature (°C)			Residue at 1000 °C (wt %)
			Onset	*T*_max_	40 wt % Loss	
CNT	0.1	0.0	495.6	520.6	591.0	48.6
	0.5	0.0	497.5	521.0	592.2	48.8
	1.0	0.0	500.7	523.7	594.3	50.6
	2.0	0.0	505.0	526.3	617.5	51.5
Hybrid	1.5	0.5	499.4	522.2	604.7	51.1
	1.0	1.0	495.2	523.4	597.7	50.9
	0.5	1.5	505.3	526.7	606.4	51.3
	0.0	2.0	506.9	529.8	612.6	50.8

**Table 4:** Glass transition temperatures for CNT series foams and Hybrid series foams obtained from the maximum of tan δ and loss modulus (*E*’’) and their corresponding intensity and full width at half maximum (FWHM).

Series	CNT (wt %)	GnP (wt %)	*T*_g_ Max tan δ (°C)	*T_g_* Max *E*’’ (°C)	tan δ Intensity	FWHM in tan δ	*E*’’ Intensity (MPa)	FWHM in *E*’’
CNT	0.1	0.0	229.1	223.5	1.56	10.6	28.1	9.0
	0.5	0.0	227.3	221.0	1.86	11.0	29.9	7.8
	1.0	0.0	227.4	220.9	1.88	11.1	43.5	7.7
	2.0	0.0	226.4	221.5	1.30	11.4	73.6	7.5
Hybrid	1.5	0.5	227.9	218.7	1.67	14.4	60.3	15.7
	1.0	1.0	228.7	220.9	1.73	12.3	54.7	10.6
	0.5	1.5	226.0	218.8	1.73	12.0	44.6	7.9
	0.0	2.0	228.6	223.3	2.03	9.5	68.4	6.1

**Table 5:** Electrical conductivity and corrected electrical conductivity values for CNT series foams and Hybrid series foams.

Series	Sample code	σ (S/m)	σ_corr_ (S/m) *
CNT	0.1 CNT	8.7 × 10^−12^	4.5 × 10^−12^
			(1.2 × 10^−12^)
	0.5 CNT	1.2 × 10^−3^	6.4 × 10^−4^
			(2.1 × 10^−4^)
	1 CNT	5.8 × 10^−3^	3.3 × 10^−3^
			(8.0 × 10^−4^)
	2 CNT	9.2 × 10^−3^	6.4 × 10^−3^
			(2.5 × 10^−3^)
Hybrid	1.5/0.5	6.9 × 10^−3^	3.7 × 10^−3^
			(1.4 × 10^−3^)
	1/1	1.6 × 10^−2^	8.8 × 10^−3^
			(3.5 × 10^−3^)
	0.5/1.5	9.6 × 10^−4^	5.9 × 10^−4^
			(1.5 × 10^−4^)
	0/2	3.9 × 10^−12^	2.2 × 10^−12^
			(6.1 × 10^−13^)

* Standard deviation of the corrected electrical conductivity is presented between parentheses.

## References

[1] Antunes M., Gedler G., Abbasi H., Velasco J.I. (2016). Graphene Nanoplatelets as a Multifunctional Filler for Polymer Foams. Mater. Today Proc..

[2] Ling J., Zhai W., Feng W., Shen B., Zhang J., Zheng W. (2013). Ge Facile Preparation of Lightweight Microcellular Polyetherimide/Graphene Composite Foams for Electromagnetic Interference Shielding. ACS Appl. Mater. Interfaces.

[3] Shen B., Zhai W., Tao M., Ling J., Zheng W. (2013). Lightweight, Multifunctional Polyetherimide/Graphene@Fe_3_O_4_ Composite Foams for Shielding of Electromagnetic Pollution. ACS Appl. Mater. Interfaces.

[4] Zhai W., Chen Y., Ling J., Wen B., Kim Y.-W. (2014). Fabrication of lightweight, flexible polyetherimide/nickel composite foam with electromagnetic interference shielding effectiveness reaching 103 dB. J. Cell. Plast..

[5] Li B., Olson E., Perugini A., Zhong W.-H. (2011). Simultaneous enhancements in damping and static dissipation capability of polyetherimide composites with organosilane surface modified graphene nanoplatelets. Polymer.

[6] Wu H., Drzal L.T. (2013). Graphene nanoplatelet-polyetherimide composites: Revealed morphology and relation to properties. J. Appl. Polym. Sci..

[7] Zhang H.-B., Yan Q., Zheng W.-G., He Z., Yu Z.-Z. (2011). Tough Graphene−Polymer Microcellular Foams for Electromagnetic Interference Shielding. ACS Appl. Mater. Interfaces.

[8] Gedler G., Antunes M., Velasco J.I., Ozisik R. (2016). Enhanced electromagnetic interference shielding effectiveness of polycarbonate/graphene nanocomposites foamed via 1-step supercritical carbon dioxide process. Mater. Des..

[9] Gedler G., Antunes M., Velasco J.I., Ozisik R. (2015). Electromagnetic shielding effectiveness of polycarbonate/graphene nanocomposite foams processed in 2-steps with supercritical carbon dioxide. Mater. Lett..

[10] Lee L.J., Zeng C., Cao X., Han X., Shen J., Xu G. (2005). Polymer nanocomposite foams. Compos. Sci. Technol..

[11] Kostopoulos V., Vavouliotis A., Karapappas P., Tsotra P., Paipetis A. (2009). Damage Monitoring of Carbon Fiber Reinforced Laminates Using Resistance Measurements. Improving Sensitivity Using Carbon Nanotube Doped Epoxy Matrix System. J. Intell. Mater. Syst. Struct..

[12] Badamshina E., Estrin Y., Gafurova M. (2013). Nanocomposites based on polyurethanes and carbon nanoparticles: Preparation, properties and application. J. Mater. Chem. A.

[13] Potts J.R., Dreyer D.R., Bielawski C.W., Ruoff R.S. (2011). Graphene-based polymer nanocomposites. Polymer.

[14] Tung V.C., Chen L.-M., Allen M.J., Wassei J.K., Nelson K., Kaner R.B., Yang Y. (2009). Low-Temperature Solution Processing of Graphene−Carbon Nanotube Hybrid Materials for High-Performance Transparent Conductors. Nano Lett..

[15] Nguyen D.D., Tiwari R.N., Matsuoka Y., Hashimoto G., Rokuta E., Chen Y.-Z., Chueh Y.-L., Yoshimura M. (2014). Low Vacuum Annealing of Cellulose Acetate on Nickel Towards Transparent Conductive CNT–Graphene Hybrid Films. ACS Appl. Mater. Interfaces.

[16] Nguyen D.D., Tai N.-H., Chen S.-Y., Chueh Y.-L. (2012). Controlled growth of carbon nanotube-graphene hybrid materials for flexible and transparent conductors and electron field emitters. Nanoscale.

[17] Kim S.H., Song W., Jung M.W., Kang M.-A., Kim K., Chang S.-J., Lee S.S., Lim J., Hwang J., Myung S. (2014). Carbon Nanotube and Graphene Hybrid Thin Film for Transparent Electrodes and Field Effect Transistors. Adv. Mater..

[18] Fan Z., Yan J., Zhi L., Zhang Q., Wei T., Feng J., Zhang M., Qian W., Wei F. (2010). A Three-Dimensional Carbon Nanotube/Graphene Sandwich and Its Application as Electrode in Supercapacitors. Adv. Mater..

[19] Deng J., Zheng R., Zhao Y., Cheng G. (2012). Vapor–Solid Growth of Few-Layer Graphene Using Radio Frequency Sputtering Deposition and Its Application on Field Emission. ACS Nano.

[20] Kim Y.-S., Kumar K., Fisher F.T., Yang E.-H. (2012). Out-of-plane growth of CNTs on graphene for supercapacitor applications. Nanotechnology.

[21] Hu Y., Li X., Wang J., Li R., Sun X. (2013). Free-standing graphene-carbon nanotube hybrid papers used as current collector and binder free anodes for lithium ion batteries. J. Power Sources.

[22] Chen S., Yeoh W., Liu Q., Wang G. (2012). Chemical-free synthesis of graphene-carbon nanotube hybrid materials for reversible lithium storage in lithium-ion batteries. Carbon.

[23] Maxian O., Pedrazzoli D., Manas-Zloczower I. (2017). Conductive polymer foams with carbon nanofillers-Modeling percolation behavior. Express Polym. Lett..

[24] Gedler G., Antunes M., Velasco J.I. (2016). Enhanced electrical conductivity in graphene-filled polycarbonate nanocomposites by microcellular foaming with sc-CO_2_. J. Adhes. Sci. Technol..

[25] Abbasi H., Antunes M., Velasco J.I. (2015). Graphene nanoplatelets-reinforced polyetherimide foams prepared by water vapor-induced phase separation. eXPRESS Polym. Lett..

[26] Abbasi H., Antunes M., Velasco J.I. (2015). Influence of polyamide-imide concentration on the cellular structure and thermo-mechanical properties of polyetherimide/polyamide-imide blend foams. Eur. Polym. J..

[27] Song P.A., Liu L., Fu S., Yu Y., Jin C., Wu Q., Li Q. (2013). Striking multiple synergies created by combining reduced graphene oxides and carbon nanotubes for polymer nanocomposites. Nanotechnology.

[28] Safdari M., Al-Haik M. (2012). Electrical conductivity of synergistically hybridized nanocomposites based on graphite nanoplatelets and carbon nanotubes. Nanotechnology.

[29] Yue L., Pircheraghi G., Monemian S.A., Manas-Zloczower I. (2014). Epoxy composites with carbon nanotubes and graphene nanoplatelets-Dispersion and synergy effects. Carbon.

[30] Li W., Xu Z., Chen L., Shan M., Tian X., Yang C., Lv H., Qian X. (2014). A facile method to produce graphene oxide-*g*-poly (l-lactic acid) as a promising reinforcement for PLLA nanocomposites. Chem. Eng. J..

[31] Sims G.L.A., Khunniteekool C. (1994). Cell-size measurement of polymeric foams. Cell. Polym..

[32] Abbasi H., Antunes M., Velasco J.I. (2018). Enhancing the electrical conductivity of polyetherimide-based foams by simultaneously increasing the porosity and graphene nanoplatelets dispersion. Polym. Compos..

[33] Huxtable S.T., Cahill D.G., Shenogin S., Xue L., Ozisik R., Barone P., Usrey M., Strano M.S., Siddons G., Shim M. (2003). Interfacial heat flow in carbon nanotube suspensions. Nat. Mater..

[34] Gupta A., Choudhary V. (2012). Effect of multiwall carbon nanotubes on thermomechanical and electrical properties of poly(trimethylene terephthalate). J. Appl. Polym. Sci..

[35] Gedler G., Antunes M., Realinho V., Velasco J.I. (2012). Thermal stability of polycarbonate-graphene nanocomposite foams. Polym. Degrad. Stab..

[36] Wang X., Yang H., Song L., Hu Y., Xing W., Lu H. (2011). Morphology, mechanical and thermal properties of graphene-reinforced poly(butylene succinate) nanocomposites. Compos. Sci. Technol..

[37] Liu L., Gu A., Fang Z., Tong L., Xu Z. (2007). The effects of the variations of carbon nanotubes on the micro-tribological behavior of carbon nanotubes/bismaleimide nanocomposite. Compos. Part A.

[38] Velasco-Santos C., Martínez-Hernández A.L., Fisher F., Ruoff R., Castano V.M. (2003). Dynamical-mechanical and thermal analysis of carbon nanotube-methyl-ethyl methacrylate nanocomposites. J. Phys. D Appl. Phys..

[39] Sung Y.T., Kum C.K., Lee H.S., Byon N.S., Yoon H.G., Kim W.N. (2005). Dynamic mechanical and morphological properties of polycarbonate/multi-walled carbon nanotube composites. Polymer.

[40] Agarwal G., Patnaik A., Sharma R.K. (2014). Mechanical and Thermo–Mechanical Properties of Bi-Directional and Short Carbon Fiber Reinforced Epoxy Composites. J. Eng. Sci. Technol..

[41] Ornaghi H.L., Bolner A.S., Fiorio R., Zattera A.J., Amico S.C. (2010). Mechanical and dynamic mechanical analysis of hybrid composites molded by resin transfer molding. J. Appl. Polym. Sci..

[42] Idicula M., Malhotra S.K., Joseph K., Thomas S. (2005). Dynamic mechanical analysis of randomly oriented intimately mixed short banana/sisal hybrid fibre reinforced polyester composites. Compos. Sci. Technol..

[43] Wu D., Lv Q., Feng S., Chen J., Chen Y., Qiu Y., Yao X. (2015). Polylactide composite foams containing carbon nanotubes and carbon black: Synergistic effect of filler on electrical conductivity. Carbon.

[44] Antunes M., Velasco J.I. (2014). Multifunctional polymer foams with carbon nanoparticles. Prog. Polym. Sci..

[45] Antunes M., Mudarra M., Velasco J.I. (2011). Broad-band electrical conductivity of carbon nanofibre-reinforced polypropylene foams. Carbon.

[46] Ameli A., Kazemi Y., Wang S., Park C.B., Pötschke P. (2017). Process-microstructure-electrical conductivity relationships in injection-molded polypropylene/carbon nanotube nanocomposite foams. Compos. Part A Appl. Sci. Manuf..

[47] Ameli A., Nofar M., Park C.B., Pötschke P., Rizvi G. (2014). Polypropylene/carbon nanotube nano/microcellular structures with high dielectric permittivity, low dielectric loss, and low percolation threshold. Carbon.

[48] Bauhofer W., Kovacs J.Z. (2009). A review and analysis of electrical percolation in carbon nanotube polymer composites. Compos. Sci. Technol..

[49] Stankovich S., Dikin D.A., Dommett G.H.B., Kohlhaas K.M., Zimney E.J., Stach E.A., Piner R.D., Nguyen S.T., Ruoff R.S. (2006). Graphene-based composite materials. Nature.

[50] Stauffer D., Aharony A. (1994). Introduction to Percolation Theory.

[51] Sahimi M. (1994). Applications of Percolation Theory.

[52] Kilbride B.E., Coleman J.N., Fraysse J., Fournet P., Cadek M., Drury A., Hutzler S., Roth S., Blau W.J. (2002). Experimental observation of scaling laws for alternating current and direct current conductivity in polymer-carbon nanotube composite thin films. J. Appl. Phys..

[53] Balberg I., Anderson C.H., Alexander S., Wagner N. (1984). Excluded volume and its relation to the onset of percolation. Phys. Rev. B.

[54] Fraysse J., Plane J. (2000). Interplay of Hopping and Percolation in Organic Conducting Blends. Phys. Status Solidi.

[55] Liu Z., Bai G., Huang Y., Ma Y., Du F., Li F., Guo T., Chen Y. (2007). Reflection and absorption contributions to the electromagnetic interference shielding of single-walled carbon nanotube/polyurethane composites. Carbon.

[56] Yang Y., Gupta M.C., Dudley K.L., Lawrence R.W. (2005). Novel Carbon Nanotube-Polystyrene Foam Composites for Electromagnetic Interference Shielding. Nano Lett..

[57] Yang Y., Gupta M.C., Dudley K.L., Lawrence R.W. (2005). A comparative study of EMI shielding properties of carbon nanofiber and multi-walled carbon nanotube filled polymer composites. J. Nanosci. Nanotechnol..

[58] Verdejo R., Bernal M.M., Romasanta L.J., Lopez-Manchado M.A. (2011). Graphene filled polymer nanocomposites. J. Mater. Chem..

